# Genome-Wide Analysis of DNA Methylation in Five Tissues of Zhikong Scallop, *Chlamys farreri*

**DOI:** 10.1371/journal.pone.0086232

**Published:** 2014-01-14

**Authors:** Yan Sun, Rui Hou, Xiaoteng Fu, Changsen Sun, Shi Wang, Chen Wang, Ning Li, Lingling Zhang, Zhenmin Bao

**Affiliations:** 1 Key Laboratory of Marine Genetics and Breeding (MGB), Ministry of Education, College of Marine Life Sciences, Ocean University of China, Qingdao, China; 2 School of Life Science, Taizhou University, Taizhou, China; Institute of Farm Animal Genetics, Germany

## Abstract

DNA methylation plays a vital role in tissue development and differentiation in eukaryotes. Epigenetic studies have been seldom conducted in the extremely diverse and evolutionarily highly successful bilaterian lineage Mollusca. In the present study, we conducted the genome-wide profiling of DNA methylation for five tissues of a bivalve mollusc, *Chlamys farreri* using the methylation-sensitive amplification polymorphism (MSAP) technique. The methylation levels were quite similar among tissues, ranging from 20.9% to 21.7%. CG methylation was the dominant type (14.9%–16.5%) in the *C. farreri* genome, but CHG methylation also accounted for a substantial fraction of total methylation (5.1%–6.3%). Relatively high methylation diversity was observed within tissues. Methylation differentiation between tissues was evaluated and 460 tissue-specific epiloci were identified. Kidney differs from the other tissues in DNA methylation profiles. Our study presents the first look at the tissue-specific DNA methylation patterns in a bivalve mollusc and represents an initial step towards understanding of epigenetic regulatory mechanism underlying tissue development and differentiation in bivalves.

## Introduction

DNA methylation is one of the key epigenetic modifications in eukaryotic genomes and is crucial for gene expression regulation during animal and plant development. In most animals studied, DNA methylation is predominantly limited to CpG doublets, which are present in substantial numbers within the CpG islands of promoters. Patterns of DNA methylation are highly variable among different animal taxa. They are fundamentally distinct between vertebrates and invertebrates [1]–[3]. Vertebrate genomes are heavily methylated at most CpGs [4], [5]. While for invertebrates, they generally exhibit diverse and complex DNA methylation patterns. For example, the ‘insect-type’ pattern shows little or no methylation in the genome (e.g., *Drosophila melanogaster* [6] and *Caenorhabditis elegans* [7]). The ‘echinoderm-type’ pattern of the sea urchin *Strongylocentrotus purpuratus* [8] carries both methylated and non-methylated fractions in the genome. The more complex pattern was found in honey bee *Apis mellifera* [9], [10], with extensive genome methylation and a fully functional set of DNA methylation enzymes [9].

In contrast to vertebrates, epigenetic studies are still lagging behind in invertebrates. Characterization of DNA methylation patterns from a broad spectrum of invertebrates would help advance the field of evolutionary epigenetics and elucidate the biological meanings of such methylation patterns. Molluscs are one of the most diverse and evolutionarily successful groups of invertebrates. They possess various body plans and adaptation strategies. Despite their species abundance and diverse geographical distribution, DNA methylation patterns in molluscs remain largely unexplored. A recent study showed that specific functional categories of genes were associated with different levels of DNA methylation in the Pacific oyster *Crassostrea gigas* [11]. For example, genes involved in stress and environmental response were prone to be hypo-methylated, which might be advantageous for greater epigenetic flexibility and for higher regulatory control [11]. Therefore, elucidating the functional significance of DNA methylation in molluscs might enhance our understanding of the epigenetic regulatory mechanisms underlying their developmental processes and environmental adaptation.

In parallel with the significance in understanding the functional roles of DNA methylation, there has been a series of methodological developments in profiling of genome-wide DNA methylation patterns. Among them, the methylation-sensitive amplification polymorphism (MSAP) technique, first described by Reyna-López et al. [12], represents a simple and cost-effective approach for genome-wide profiling of DNA methylation without requiring any genome information. It utilizes the restriction isoschizomer pair *Hpa*II and *Msp*I, which differs in their sensitivity to the methylation state of their recognition site 5′-CCGG-3′, thereby allowing large-scale interrogating the methylation states of all or a subset of CCGG regions across the genome. MSAP combines the features of amplified fragment length polymorphism (AFLP) assay with methylation-sensitive restriction enzymes, and has proven to be highly efficient for large-scale detection of cytosine methylation in the genomes of both model and non-model organisms [13]–[17]. Recently, MSAP has also been utilized in ecological studies to help understand the impacts of epigenetic processes in an ecological context [18], [19]. Despite its wide utilization in epigenetic studies, scoring and analysis of MSAP data that contain multistate information (summarized in Table 1) is not straightforward. Many scoring approaches have been proposed for MSAP analyses, but estimates in epigenetic diversity and differentiation may vary strongly between different methods [20]. According to a recent study by Schulz et al. [20], the use of ‘Mixed Scoring 2’ approach is recommended as it separately takes into account different methylation types and enables more detailed estimation of epigenetic diversity.

**Table 1:** MSAP-profiled methylation types and the scoring scheme.

Type	Restriction pattern		Methylation status^c^	Scoring scheme			
	H^a^	M		unmethylated	^HMe^CG/^Me^CG	^HMe^CCG	
I	+^b^	+	unmethylated	1	0	0	
II	–	+	^HMe^CG/^Me^CG	0	1	0	
III	+	–	^HMe^CCG	0	0	1	
IV	–	–	^Me^CCGG/^HMe^C^HMe^CGG/^Me^C^Me^CGG/mutation	0	0	0	

^a^ H and M indicate the enzyme combinations of *Eco*RI/*Hpa*II and *Eco*RI/*Msp*I;

^b^+: band presence; −: band absence;

c HMe: hemi-methylated on only one strand; Me: fully methylated on both strands.

*Chlamys farreri* (Jones et Preston 1904), also known as Zhikong scallop, naturally inhabits along the seacoasts of China, Japan and Korea and is one of the most important maricultural shellfish in northern China. Although it has been extensively studied at the molecular level, its epigenetic modification patterns have not been investigated yet. In the present study, we performed genome-wide DNA methylation profiling in five tissues of *C. farreri* using the MSAP technique to investigate the epigenetic diversity and differentiation among tissues.

## Results

### Interrogating the Methylation Status of CCGG Sites using MSAP

The isoschizomers *Hpa*II and *Msp*I recognize and cleave the same 5′-CCGG-3′ sites, but differ in their sensitivity to the methylation state of cytosine. *Hpa*II is inactive if either cytosine is fully methylated (both strands are methylated), but it cleaves the hemi-methylated sequence when the external cytosine on only one strand is methylated (^HMe^CCG). *Msp*I is sensitive only to methylation at the internal cytosine; it cuts when the inner cytosine is methylated (^HMe^CG or ^Me^CG) but not the outer one. Sites that are fully methylated at the external cytosine (^Me^CCG) or hemi- or fully methylated at both internal and external cytosines (^HMe^C^HMe^CG or ^Me^C^Me^CG) are not cut by either enzyme. Therefore, comparison of the *Eco*RI/*Hpa*II and *Eco*RI/*Msp*I profiles allows the detection of the methylation state at a site.

**Figure 1:**
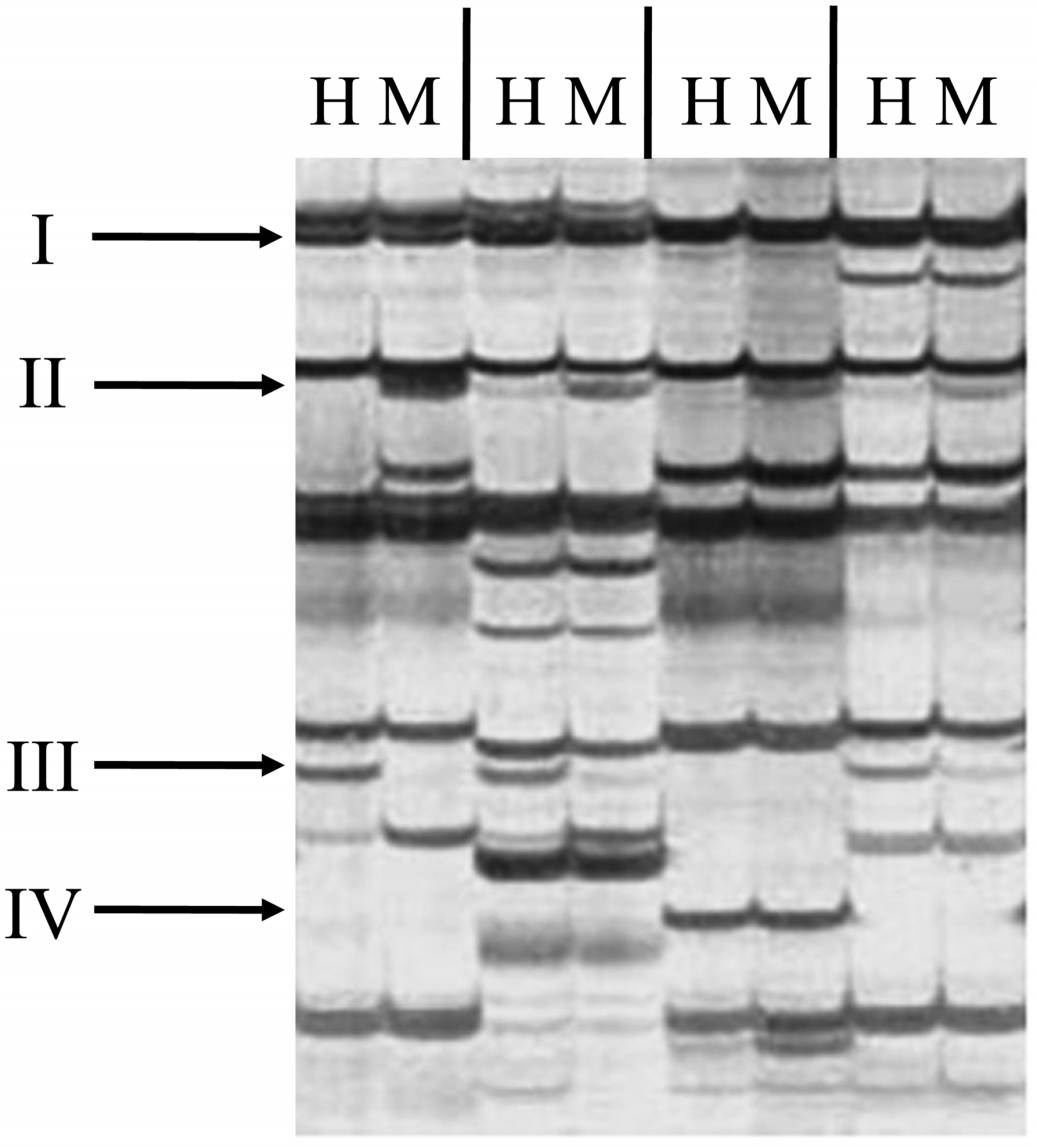
An example of DNA methylation profiles in *C. farreri*. H and M indicate the enzyme combinations of *Eco*RI/*Hpa*II and *Eco*RI/*Msp*I.

Depending on the presence or absence of bands in the *Hpa*II and *Msp*I panels, four types can be distinguished (Table 1): Type I, presence of bands in both panels indicates an unmethylated state; Type II, presence of bands only in the *Msp*I panel indicates ^HMe^CG or ^Me^CG sites; Type III, presence of bands only in the *Hpa*II panel indicates ^HMe^CCG sites; Type IV, absence of bands in both panels represents an uninformative state as multiple reasons can account for this (e.g., ^Me^CCG or ^HMe^C^HMe^CG or ^Me^C^Me^CG sites or a mutation). Examples of the different methylation types are shown in Figure 1.

### Global Methylation Levels in Five Tissues of *C. farreri*

DNA methylation profiles were generated for five tissues of six scallop individuals based on MSAP analysis. To obtain large-scale DNA methylation profiles, a total of 20 selective primer pairs were utilized in the MSAP procedure by combining five *Eco*RI primers (E32, E35, E38, E39 and E45) with four *Hpa*II/*Msp*I primers (HM1−4). In total, 1,367 CCGG sites were detected across all samples, of which, an average of 124 and 47 was detected as type II and type III methylation sites, respectively. On average, 824, 763, 812, 811 and 807 sites were detected for gill (G), kidney (K), ovary (O), smooth muscle (Sm) and striated muscle (St), respectively. The average number of sites detected in K was significantly (*P*<0.05) lower than that detected in the other tissues, indicating more type IV methylation sites, possibly caused by ^Me^CCGG, ^HMe^C^HMe^CGG or ^Me^C^Me^CGG, exist in K than the other tissues. Similar number of type II and type III methylation sites was found in different tissues. Specifically, 125, 126, 121, 122 and 123 sites on average were detected as type II methylation sites and 40, 48, 51, 50 and 46 sites as type III methylation sites for G, K, O, Sm and St, respectively.

**Figure 2:**
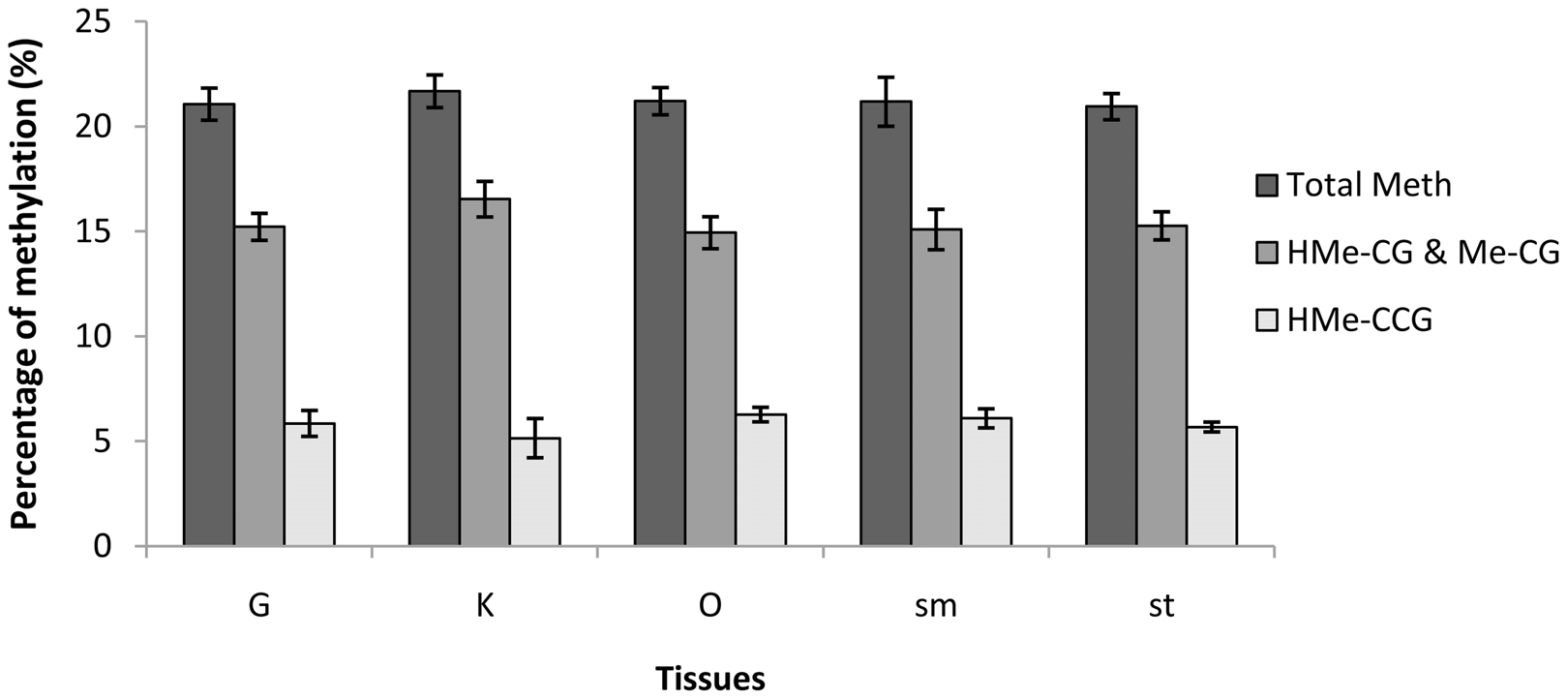
DNA methylation levels in five tissues of *C. farreri*. Methylation levels are presented as Mean±SEM.

Global DNA methylation level was estimated for each tissue by dividing the number of type II and type III sites by the number of type I, type II and type III sites. It turned out that global methylation levels were quite similar across tissues, with averages of 21.06%, 21.68%, 21.20%, 21.18% and 20.94% for G, K, O, Sm and St, respectively (Figure 2). Type II methylation (i.e., ^HMe^CG or ^Me^CG) was the most dominant type in the *C. farreri* genome, with average levels of 15.22%, 16.53%, 14.93%, 15.09% and 15.27% for G, K, O, Sm and St, respectively. Type III methylation (i.e., ^HMe^CCG) also accounted for a substantial fraction of total methylation, with average levels of 5.85%, 5.14%, 6.27%, 6.09% and 5.68% for G, K, O, Sm and St, respectively. No significant difference was detected in global, type II or type III methylation levels between tissues.

### Epigenetic Diversity within Tissues

The multistate raw data matrix resulting from the *Eco*RI/*Hpa*II and *Eco*RI/*Msp*I profiles needs to be transformed into a binary data matrix before statistical analyses or computation of descriptive indices such as epigenetic diversity. Here, ‘Mixed Scoring 2’ approach [20] was adopted, which extracts three epiloci (unmethylated, ^HMe^CG & ^Me^CG, ^HMe^CCG) from each CCGG site and thus enables more detailed estimation of epigenetic diversity.

**Figure 3:**
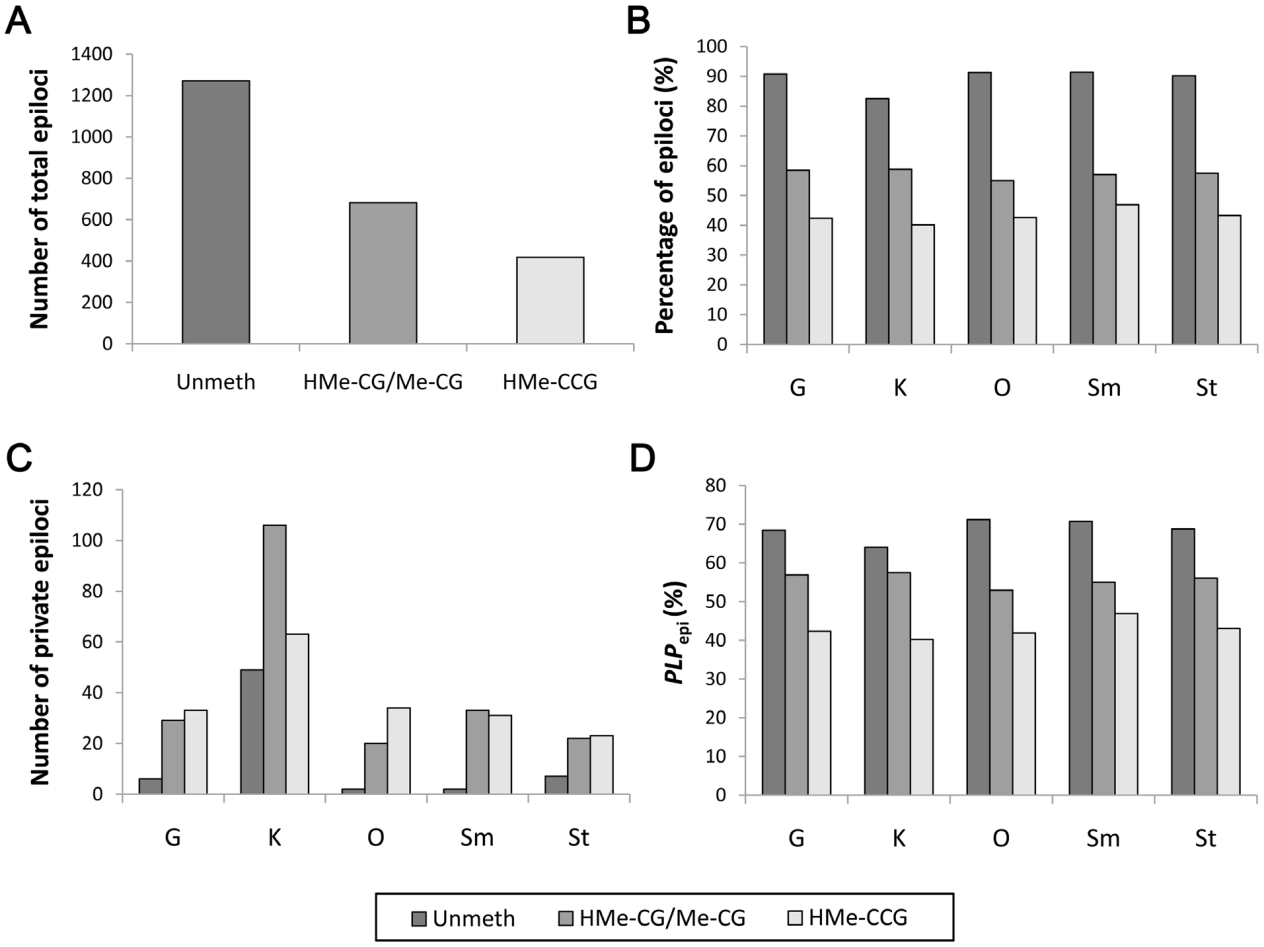
Epigenetic diversity in five tissues of *C. farreri*. (A) Total number of three epiloci types; (B) Percentage of three epiloci types in each tissue; (C) Number of private epiloci in each tissue; (D) Percentage of polymorphic epiloci (*PLP*_epi_) for each epoloci type in the five tissues.

Of the 1,367 CCGG sites, 184 were monomorphic across all tissues and individuals, accounting for 13% of total sites. After binary data transformation, 2,371 epiloci were obtained from the raw MSAP data, consisting of 1,271, 682 and 418 epiloci for the three epiloci types (Table 2, Figure 3A). The most abundant epiloci type in the five tissues was unmethylated type (83% to 91%), followed by ^HMe^CG/^Me^CG type (55% to 59%) and ^HMe^CCG type (40% to 47%) (Table 2, Figure 3B).

**Table 2:** Summary of epigenetic diversity in five tissues of *C. farreri.*

Type of loci	Tissue	Total epiloci	Number/percentage(%) of epiloci^a^	Number/percentage(%) of private epiloci	Number ofpolymorphic epiloci	*PLP*_epi_ b (%)
Unmethylated	G	1271	1154/90.79	6/0.47	870	68.45
	K	1271	1049/82.53	49/3.86	814	64.04
	O	1271	1161/91.35	2/0.16	905	71.20
	Sm	1271	1162/91.42	2/0.16	899	70.73
	St	1271	1147/90.24	7/0.55	874	68.76
^HMe^CG/^Me^CG	G	682	399/58.50	29/4.25	388	56.89
	K	682	401/58.80	106/15.54	392	57.48
	O	682	375/54.99	20/2.93	361	52.93
	Sm	682	389/57.04	33/4.84	375	54.99
	St	682	392/57.48	22/3.23	382	56.01
^HMe^CCG	G	418	177/42.34	33/7.89	177	42.34
	K	418	168/40.19	63/15.07	168	40.19
	O	418	178/42.58	34/8.13	175	41.87
	Sm	418	196/46.89	31/7.42	196	46.89
	St	418	181/43.30	23/5.50	180	43.06

a Number of epiloci: number of epiloci present (with at least one “1”-score) per tissue;

b *PLP*_epi_: percentage of polymorphic epiloci.

Of the 2,371 epiloci, 2,187 were polymorphic across all samples, consisting of 1,089, 680 and 418 epiloci for the three epiloci types. High percentage of polymorphic epiloci were observed for all three epiloci types (Table 2, Figure 3D), with the unmethylated type being the highest (64% to 71%), followed by the ^HMe^CG/^Me^CG type (53% to 57%) and ^HMe^CCG type (40% to 47%). K showed the highest epiloci polymorphism (57%) for the ^HMe^CG/^Me^CG type but the lowest epiloci polymorphism (64% and 40%) for the unmethylated and ^HMe^CCG types. O showed the highest epiloci polymorphism (71%) for the unmethylated type but the lowest epiloci polymorphism (53%) for the ^HMe^CG/^Me^CG type. Sm showed the highest epiloci polymorphism (47%) for the ^HMe^CCG type.

### Epigenetic Differentiation among Tissues

**Figure 4:**
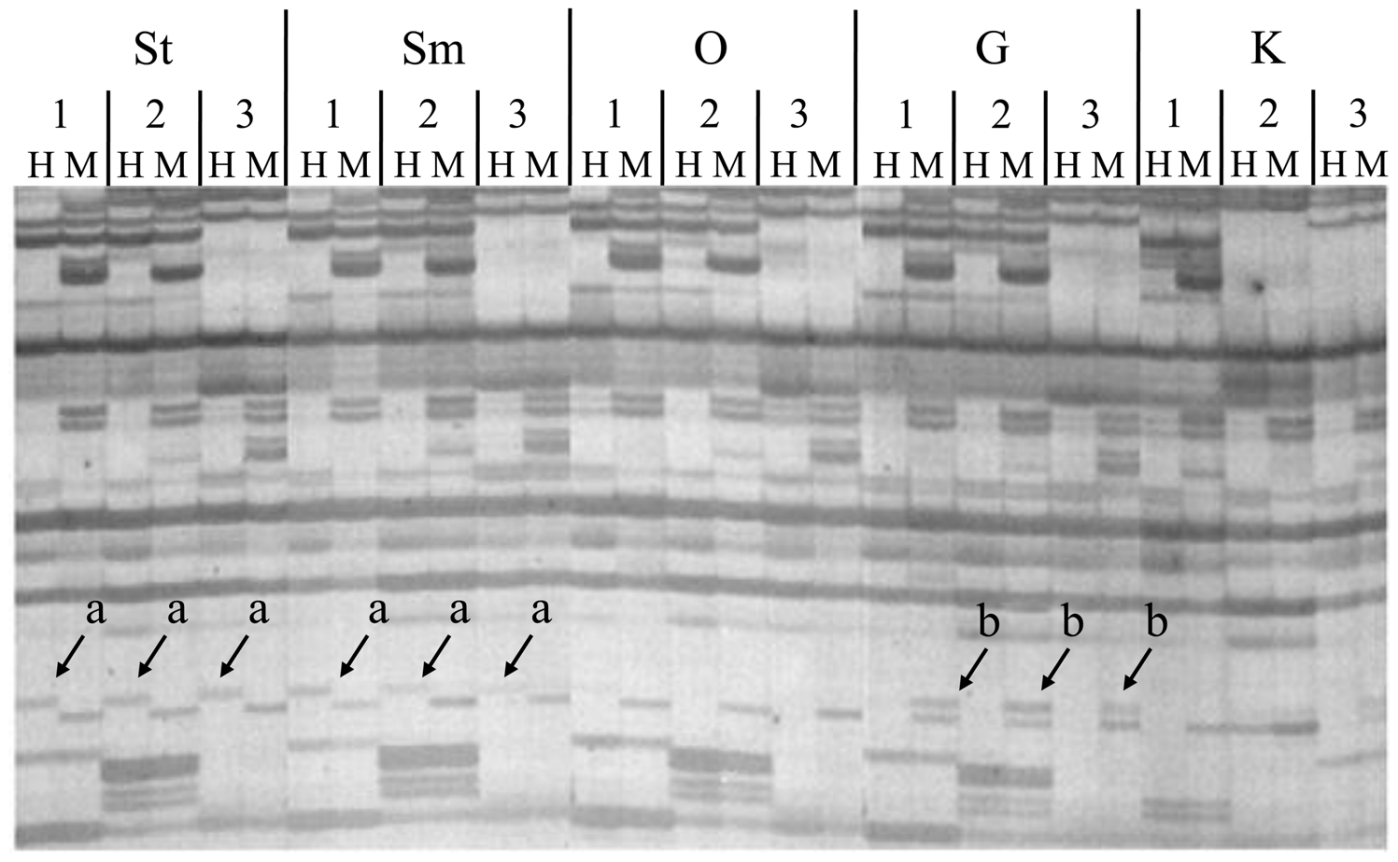
An example of private epiloci detected by MSAP analysis of three *C. farreri* individuals. a, ^HMe^CCG site specifically detected in striated and smooth muscle; b, ^HMe^CG/^Me^CG site specifically detected in gill.

To evaluate epigenetic differentiation among tissues, we first searched for private epiloci (i.e., tissue-specific epiloci, Figure 4) for each of the five tissues. In total, 460 private epiloci were identified, of which 66, 210 and 184 belonged to the unmethylated, ^HMe^CG/^Me^CG and ^HMe^CCG type, respectively, suggesting that ^HMe^CG/^Me^CG and ^HMe^CCG were two dominant private epiloci types. When looking at the tissue level, there were 68, 218, 56, 66 and 52 private epiloci in G, K, O, Sm and St, respectively. The total number of private epiloci in K significantly exceeded that in the other tissues (*P*<0.05) (Table 2, Figure 3C). More strikingly, both ^HMe^CG/^Me^CG and ^HMe^CCG private epiloci in K accounted for more than 15% of the total number of the corresponding epiloci type.

**Figure 5:**
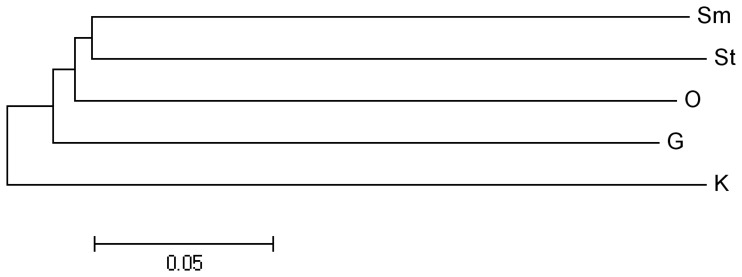
Epigenetic relationships of five tissues of *C. farreri*.

Epigenetic differentiation among tissues was further evaluated by calculating pairwise epi-distances between tissues (Table 3, Figure 5) to form an overview of epigenetic relationships of the five tissues. As expected, Sm and St showed the highest epigenetic similarity (0.66) and thus were clustered together first (Figure 5). O showed higher similarity to Sm and St than to G and K. Consistent with the private epiloci analysis, K seems to be the most divergent tissue as it also showed high epi-distances to all the other tissues.

**Table 3:** Methylation differentiation among five tissues of *C. farreri.*

	G	K	O	Sm	St
G	–	0.622	0.658	0.653	0.645
K	0.378	–	0.615	0.613	0.611
O	0.342	0.385	–	0.660	0.655
Sm	0.347	0.387	0.340	–	0.661
St	0.355	0.389	0.345	0.339	–

Above/below the diagonal: epigenetic similarities/distances.

## Discussion

Cytosine methylation arises from the addition of a methyl group to a cytosine’s C5 carbon residue, which can occur in different sequence contexts. In higher plants, DNA methylation can occur at cytosines in both symmetric sequence contexts of CG and CHG (H = A, T or C), and also in an asymmetric CHH context. Differing from plants, DNA methylation in mammals was once thought to occur almost exclusively in the symmetric CG context. However, recent methylome studies are challenging this perception by discovering the prevalence of non-CG methylation in human embryonic stem (ES) cells, e.g., nearly 25% of all methylated cytosines in the human ES cells did not occur exclusively at CG sites [21], [22]. Non-CG methylation also exists in many invertebrates such as *Ciona intestinalis*, *Apis mellifera*, *Tribolium castaneum*, *Bombyx mori*, *Drosophila melanogaster* and *Nematostella vectensis*, but is usually present at much low levels in the genome (0.22–1.65% for CHG and 0.22–1.31% for CHH) [23]. MSAP technique can detect the hemi-methylation at the external cytosine of CCGG sites (i.e., ^HMe^CCG), and therefore, to some extent, provides a way to evaluate non-CG methylation in the genome. According to our study, CG methylation is the dominant methylation type in the *C. farreri* genome, but a substantial amount of DNA methylation is derived from CHG methylation (5.1%–6.3%). Such high CHG methylation is unexpected, and whether this observation applies to other molluscs remains to be tested; but it is foreseeable that understanding of the biological significance of non-CG methylation in molluscs represents an exciting area for further research.

DNA methylation is essential for tissue development and differentiation. Using MSAP technique, many studies have revealed differential DNA methylation levels among tissues in different organisms. For instance, DNA methylation level ranges from 20.24% to 21.78% in maize [24], from 26.1% to 29.4% in chicken [25] and from 50.18% to 53.99% in swine [16]. In this study, quite similar methylation levels (20.9–21.7%) were found across the five tissues of *C. farreri*, and no significant difference was detected between different tissues. The methylation variation among different tissues of *C. farreri* (0.74%) is relatively smaller in comparison with the aforementioned organisms (1.54%–3.81%), suggesting that methylation level might be less divergent among tissues in scallops. But it should be noted that the actual methylation level of *C. farreri* might be underestimated due to the following reasons. First, MSAP technique can only interrogate the methylation status of CG and CHG sites, leaving CHH sites undetected. Second, type IV methylation (e.g., ^Me^CCG, ^HMe^C^HMe^CG or ^Me^C^Me^CG) is also excluded from the calculation of total methylation level because the same *Hpa*II/*Msp*I profiles can also be caused by a mutation at the restriction site.

Our MSAP analysis revealed relatively high methylation diversity within tissues, with 64% to 71% for the unmethylated epiloci type, 53% to 57% for the ^HMe^CG/^Me^CG epiloci type and 40% to 47% for the ^HMe^CCG epiloci type. The high methylation diversity is unlikely to be affected by environmental effects as all assayed individuals were reared in the same lantern net. MSAP analysis of another cohort of individuals also revealed similar level of methylation diversity (data not shown). There are several possible causes may account for this observation. First, the high methylation diversity may be related to the high genome heterozygosity of *C. farreri* (∼1.4%) [26]. Recent studies have shown that genetic variation may have a substantial impact on the local methylation patterns [27]–[30]. For example, a large-scale association analysis in humans demonstrated a strong genetic component in DNA methylation profiles [31]. It is therefore possible that high genome heterozygosity can lead to high methylation diversity by changing local methylation patterns. Second, DNA demethylation may be another possible cause accounting for high methylation diversity. DNA demethylation plays a pivotal role in shaping methylation patterns, but the underlying mechanisms are still incompletely understood [32]–[34]. The process of DNA demethylation can be passive or active, entailed by the ten-eleven translocation (TET) and AID/APOBEC families of enzymes. Therefore, dynamics of this process could also lead to the observed epigenetic diversity.

Although no significant difference in methylation levels was detected between tissues of *C. farreri*, our study identified 460 private epiloci, of which 68, 218, 56, 66 and 52 were from tissue G, K, O, Sm and St, respectively. These private epiloci may be related to tissue development and differentiation, and thus constitute a core set of epi-marker resource that would facilitate further epigenetic studies in this species. Analysis of epigenetic relationships of the five tissues revealed the highest epigenetic similarity (0.66) between Sm and St, which seems reasonable because these two tissues perform quite similar biological functions. However, K seems to be quite different from the other tissues in DNA methylation profiles. It possesses the most abundant private epiloci and shows the lowest similarity to all the other tissues. Why K established a relatively unique DNA methylation profile is not clear and is worthy of further investigation.

## Conclusions

In the present study, genome-wide profiling of DNA methylation was conducted for five tissues of *C. farreri* using MSAP technique. Methylation diversity within tissues and differentiation between tissues were evaluated. A substantial amount of tissue-specific epiloci was identified. Kidney differs from the other tissues in DNA methylation profiles. Our study presents the first look at the tissue-specific DNA methylation patterns in a bivalve mollusc and represents an initial step towards understanding of epigenetic regulatory mechanism underlying tissue development and differentiation in bivalves.

## Materials and Methods

### Ethics Statement

All scallop handling was conducted in accordance with the guidelines and regulations established by the Ocean University of China and the local government.

### Scallop Sample Collection and DNA Isolation

A cohort of two-year-old *C. farreri* individuals were obtained from the hatchery of Xunshan Group (Shandong province, China). Six female individuals were chosen to measure epigenetic diversity within tissues and evaluate epigenetic differentiation between tissues. Individuals used in the MSAP analysis were all collected from the same lantern net to minimize the possible environmental effects on the inferred methylation patterns. Five tissues (gill, kidney, ovary, smooth muscle and striated muscle) were dissected from the sampled individuals and stored at −80°C for further analysis. Genomic DNA was isolated from each tissue using the traditional phenol/chloroform extraction method [35].

### MSAP Procedure

MSAP experiments were performed according to the Xiong’s protocol [13] with minor modifications. DNA samples were separately digested with the enzyme combinations of *Eco*RI/*Hpa*II and *Eco*RI/*Msp*I (TaKaRa, Dalian, China). The digestion reaction was performed in a volume of 20 µL containing 200 ng DNA template, 10 U *Eco*RI and 10 U *Hpa*II (or *Msp*I). The reaction mixture was incubated at 37°C for 3 h and then inactivated at 65°C for 10 min. The ligation reaction was performed in a volume of 25 µL containing 20 µL digestion mixture, 5 U T4 DNA ligase (TaKaRa, Dalian, China), 10 pmol *Eco*RI adapter, 50 pmol *Hpa*II/*Msp*I adapter and 1×T4 ligation buffer. The ligation mixture was incubated at 16°C for 12–16 h. Pre-amplification reaction was performed in a volume of 50 µL containing 5 µL of ligation product, 75 ng of each pre-amplification primer (E00+A and HM00+T, Table S1), 1 U Taq polymerase, 0.2 mM each dNTP, 1.5 mM MgCl_2_ and 1×PCR buffer. Pre-amplifications were programmed as 25 cycles of 94°C for 90 s, 56°C for 30 s and 72°C for 1 min. Selective amplification reaction was performed in a volume of 20 µL containing 5 µL of 20-fold diluted pre-amplification product, 30 nM of each selective-amplification primer (Table S1), 0.5 U Taq polymerase, 0.2 mM each dNTP, 1.5 mM MgCl_2_ and 1×PCR buffer. Selective amplifications were programmed as: 13 cycles of 94°C for 30 s, 65°C for 30 s (reduced by 0.7°C each cycle) and 72°C for 1 min; followed by 23 cycles of 94°C for 30 s, 56°C for 30 s and 72°C for 1 min.

### Detection Assay

The products of selective amplifications were separated by 4.5% denaturing polyacrylamide gel electrophoresis at 60 W for 1.5 h and PCR fragments were visualized by silver staining [36]. The electrophoretic images were scanned and clear and unambiguous bands were used for further analysis.

### MSAP Data Analysis

MSAP data were scored using the ‘Mixed Scoring 2’ approach as recommended by Schulz et al. [20], which converts raw data into three epiloci types (Table 1). For unmethylated sites, ^HMe^CG- or ^Me^CG-sites, and ^HMe^CCG-sites, type I, II and III was scored as ‘1’, respectively. Type IV was always scored as ‘0’. The program MSAP_calc [20] offers functions to transform MSAP raw data into binary epigenetic loci and to calculate descriptive parameters (e.g., total number of epiloci, and numbers of private and polymorphic epiloci) of epigenetic variation using the R environment. Methylation differentiation among five tissues was measured by calculating pairwise epi-distances between tissues (i.e., 1 minus epi-similarity).

## Supporting Information

Table S1 MSAP adapter and primer sequences.
(PDF)
