# Supplementary material for: Genome-Wide Analysis of DNA Methylation in Five Tissues of Zhikong Scallop, Chlamys farreri
Source: PLoS One. 2014 Jan 14;9(1):e86232. doi: 10.1371/journal.pone.0086232 (PMC3891877; doi:10.1371/journal.pone.0086232)
Supplement: Table S1 — MSAP adapter and primer sequences. (PDF) [file pone.0086232.s001.pdf]

Table S1 Adapter and primer sequences

Adapter/ Primer		Sequence (5' to 3')
*EcoR*I adapter		5'-CTCGTAGACTGCGTACC-3'
		3'-CTGACGCATGGTTAA-5'
E00		GACTGCGTACCAATTC
PreAmp primer: E00+A		GACTGCGTACCAATTCA
Selective primers:	E00+AAC (E32)	GACTGCGTACCAATTCAAC
	E00+ACA (E35)	GACTGCGTACCAATTCACA
	E00+ACT (E38)	GACTGCGTACCAATTCACT
	E00+AGA (E39)	GACTGCGTACCAATTCAGA
	E00+ATG (E45)	GACTGCGTACCAATTCATG
*Hpa*II/ *Msp*I adapter		5'-GACGATGAGTCTAGAA-3'
		3'-CTACTCAGATCTTGC-5'
HM00		GATGAGTCTAGAACGG
PreAmp primer: HM00+T		GATGAGTCTAGAACGGT
Selective primers:	HM00+TAC (H1)	GATGAGTCTAGAACGGTAC
	HM00+TAG (H2)	GATGAGTCTAGAACGGTAG
	HM00+TTC (H3)	GATGAGTCTAGAACGGTTC
	HM00+TTG (H4)	GATGAGTCTAGAACGGTTG
